# Understanding Thermostability Factors of Barley Limit Dextrinase by Molecular Dynamics Simulations

**DOI:** 10.3389/fmolb.2020.00051

**Published:** 2020-04-16

**Authors:** Juan Du, Jianjun Dong, Songjie Du, Kun Zhang, Junhong Yu, Shumin Hu, Hua Yin

**Affiliations:** ^1^State Key Laboratory of Biological Fermentation Engineering of Beer, Tsingtao Brewery, China; ^2^Shandong Province Key Laboratory of Applied Mycology, College of Life Sciences, Qingdao Agricultural University, Qingdao, China

**Keywords:** barley limit dextrinase, thermostability, molecular dynamics simulation, hydrogen bond, salt-bridge

## Abstract

Limit dextrinase (LD) is the only endogenous starch-debranching enzyme in barley (*Hordeum vulgare*, *Hv*), which is the key factor affecting the production of a high degree of fermentation. Free LD will lose its activity in the mashing process at high temperature in beer production. However, there remains a lack of understanding on the factor affecting the themostability of *Hv*LD at the atomic level. In this work, the molecular dynamics simulations were carried out for *Hv*LD to explore the key factors affecting the thermal stability of LD. The higher value of root mean square deviation (RMSD), radius of gyration (*R*_g_), and surface accessibility (SASA) suggests the instability of *Hv*LD at high temperatures. Intra-protein hydrogen bonds and hydrogen bonds between protein and water decrease at high temperature. Long-lived hydrogen bonds, salt bridges, and hydrophobic contacts are lost at high temperature. The salt bridge interaction analysis suggests that these salt bridges are important for the thermostability of *Hv*LD, including E568–R875, D317–R378, D803–R884, D457–R214, D468–R395, D456–R452, D399–R471, and D541–R542. Root mean square fluctuation (RMSF) analysis identified the thermal-sensitive regions of *Hv*LD, which will facilitate enzyme engineering of *Hv*LD for enhanced themostability.

## Introduction

Limit dextrinase (LD), also termed R-enzyme, pullulanase, isoamylase, or amylopectin 6-glucanohydrolase, is the only endogenous starch-debranching enzyme in barley (*Hordeum vulgare*, *Hv*) that digests amylopectin and dextrins (Manners et al., 1970; Yang et al., 2008). *Hv*LD belongs to the glycoside hydrolase family 13 subfamily 13 (GH13_13) and can cleave α-1,6-glucosidic bonds in limit dextrins derived from amylopectin (Stam et al., 2006).

Barley is a major raw material in beer production. The major biochemical process in brewing is to degrade barley starch into fermentable sugars, which are further converted into alcohol by yeast metabolism. *Hv*LD as a specific enzyme to digest amylopectin and dextrins is the key factor affecting the production of a high degree of fermentation (Wang et al., 2015). There are three different forms of LD existing in barley: insoluble bound, soluble inactive, and active free. Only the free form is capable for degrading amylopectin (Sissons et al., 1994). The essential industrial process of brewing includes three steps: malting, mashing, and fermentation. Mashing is usually performed at 60–70°C and at a pH of below 4.5 (Moshi et al., 2015). The heat resistance of LD in free form is poorer than the bound or latent form (Sissons et al., 1995). Free LD will lose its activity in the mashing process at a temperature higher than 63°C (Sissons et al., 1995). High themostability and activity of LD is desirable for the beer production.

**FIGURE 1:**
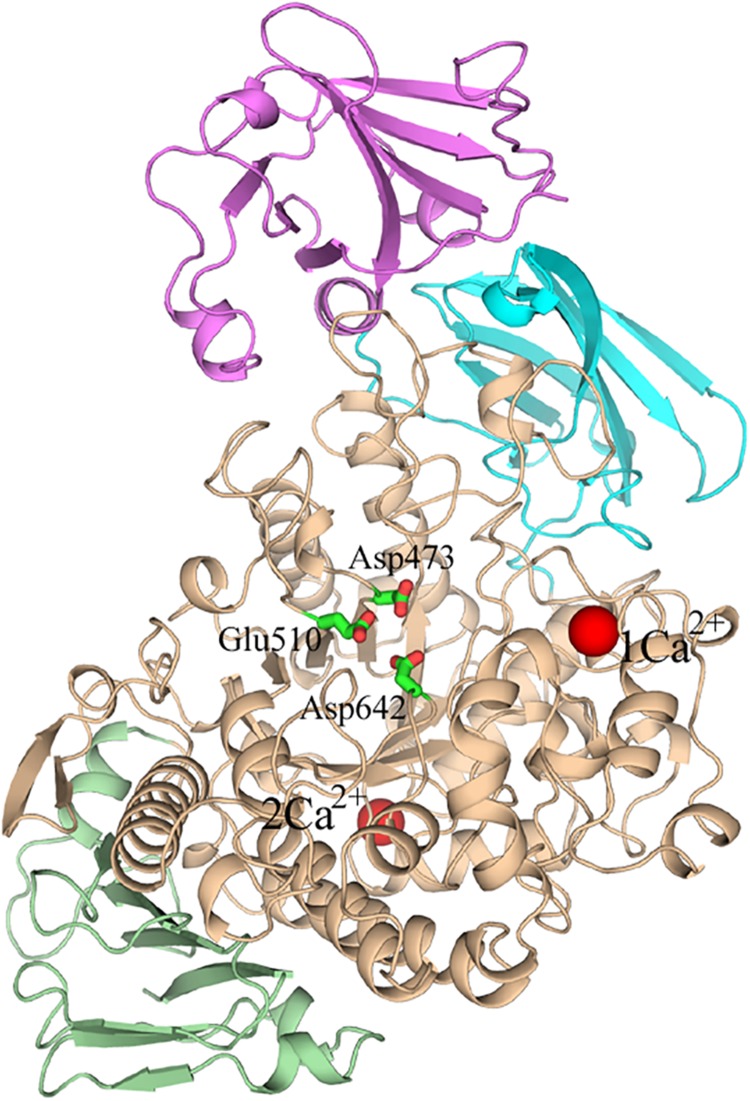
The overall structure of Barley limit dextrinase. N-domain, Magenta; CBM48, cyan; catalytic domain, wheat; C-domain, light green; Ca^2+^ ions, red spheres. The catalytic residues (Asp473, Glu510, and Asp642) are represented in sticks.

Several crystal structures of *Hv*LD have been reported (Vester-Christensen et al., 2010; Møller et al., 2012a, Møller et al., 2015a, b). The *Hv*LD structure contains four domains (Vester-Christensen et al., 2010) (shown in Figure 1): the N-terminal domain, a carbohydrate binding module 48 (CBM48), a catalytic (β/α) 8 domain containing the two catalytic residues (Asp473, nucleophile; Glu510, general acid/base) and the transition-state stabilizer (Asp642), and a C-terminal domain. The N-terminal domain includes residues 2–124 resembling carbohydrate binding module 21. CBM48 includes residues 125–230. The catalytic domain contains residues 231–774 and the C-terminal domain contains residues 775–885.

There are several works focused on improvement of the themostability of pullulanase derived from bacteria (Chen et al., 2015; Li et al., 2015; Chang et al., 2016; Wang et al., 2016). However, it is still lacking the understanding on the factor affecting the themostability of *Hv*LD at the atomic level. It is suggested that enzymes keep their structural stability by various kinds of non-covalent interactions, such as hydrogen bonds, salt bridges, disulfide bonds, and hydrophobic interaction (Nick Pace et al., 2014; Nilofer et al., 2017). Recently, molecular dynamics (MD) simulation, as a useful tool, has been widely applied to find important characteristics of protein stability (Alizadeh-Rahrovi et al., 2015; Sharma and Sastry, 2015; Jiang et al., 2016; Idrees et al., 2017; Gu et al., 2019).

In this work, MD simulations were carried out for barley limit dextrinase (*Hv*LD) to explore the key factors affecting the thermal stability of LD. The root mean square deviation (RMSD), radius of gyration (*R*_g_), and surface accessibility (SASA) were calculated to explore the dynamics of *Hv*LD. Intra-protein hydrogen bonds, protein–water hydrogen bonds, salt bridges, and hydrophobic interaction were analyzed to find the factors about the thermal stability of *Hv*LD. Finally, root mean square fluctuation (RMSF) analysis was performed to identify the thermal-sensitive regions of LD. The structural and dynamic details will help to understand the driving forces that lead to the stability of *Hv*LD at different temperatures, which will facilitate enzyme engineering of *Hv*LD.

## Materials and Methods

### Systems Preparation

The X-ray structure of barley LD (PDB ID: 4CVW) (Møller et al., 2015a) was obtained from the RCSB Protein Data Bank. The LD inhibitor was removed from this structure. The structure of barley LD (PDB ID: 4CVW) was superimposed on the free form of HvLD (PDB ID: 4AIO) (Møller et al., 2012b). The missing residues (43-PSN-45, 102-FGADGK-107) were also built based on the coordinate of the corresponding residues in the free form of HvLD (PDB ID: 4AIO). A mutant of LD^D317A^ was constructed to evaluate the effect of salt bridge between Asp317 and Arg378.

### MD Simulations

Standard AMBER ff03 force field (Wang et al., 2004; Hornak et al., 2006) was assigned to the protein. The force field parameter developed by Bradbrook et al. (1998) was assigned for the Ca^2+^. The protonation state of ionizable residues was set under pH 5.5 based on the pKa values calculated by the H++ server (Anandakrishnan et al., 2012). Na^+^ ions were added to neutralize the overall system. Each system was embedded in a rectangular box of the TIP3P water molecule (Jorgensen et al., 1983), maintaining a distance of 10 Å from any solute atom to the boundary.

The MD simulations were performed using AMBER12. Energy minimization was carried out with a decreasing harmonic force constraint on the protein. The minimized system was gradually heated from 0 K to the desired temperature within 200 ps under the NVT ensemble condition. The temperature was set as 298 K, 318 K (optimum temperature), and 343 K (the highest mashing temperature), respectively. To investigate the effect of calcium ions for the structural stability, three systems without Ca^2+^ at 298, 318, and 343 K were also constructed. The temperature was set as 298 and 343 K for LD^D317A^. Then, the system was relaxed within 1.55 ns under the *NPT* ensemble condition. Finally, a total of 100 ns was simulated to produce trajectories under the *NPT* ensemble condition for each system. A 50-ns MD simulation was conducted for LD^D317A^ at both temperatures. The covalent bonds to hydrogen atoms were constrained using the SHAKE algorithm (Coleman et al., 1977) and the Particle Mesh Ewald (PME) method (Darden et al., 1993) was employed to calculate long-range electrostatic interactions. The real space cutoff was set at 10.0 Å, the same as that for van der Waals interactions. The grid-spacing and convergence criteria of PME calculation was set to 1 Å and 1.0E-05, respectively. The time step used for the simulations was set to 2 fs. The atom coordinates were saved every 10 ps for subsequent analysis.

### Analysis

All of the analyses were performed using the analytical tools cpptraj (Roe and Cheatham, 2013) module in AMBER tools and VMD (Humphrey et al., 1996). The RMSD was calculated as the deviation between backbone atoms of the protein with respect to the initial structure’s backbone atoms, averaged over the backbone atoms. For the RMSF and SASA, the average value was calculated over time. SASA was calculated for all of the residues, hydrophobic residues, hydrophilic residues, and catalytic residues (Asp473, Glu510, and Asp642), respectively. The *R*_g_ was calculated by VMD.

The hydrogen bond was calculated based on a maximum cutoff distance between the donor and the acceptor at 3.5 Å and the angle of donor–hydrogen–acceptor larger than 120°. The average value of the number of hydrogen bond was calculated as the ratio of the sum of the total number of HBs in each frame to the total number of frames. The redundant hydrogen bonds between the same donor and acceptor but with different hydrogen atoms were removed, saving the one with the highest occupancy. The HBs were analyzed by considering the chemical properties of different residues, including charged residues (Arg, Lys, Asp, and Glu), polar residues (Gln, Asn, Ser, Thr, Tyr, and Cys), and hydrophobic residues (Ala, Ile, Leu, Phe, Val, Pro, Gly, Met, and Trp).

The salt bridges (SBs) were considered to be formed if the distance between an oxygen atom of an acidic residue (Oδ1 and Oδ2 of ASP and Oδ1 and Oδ2 of Glu) and the nitrogen atom of a basic residue (Nε, Nη1 and Nη2 of Arg and Nζ of Lys) was less than 4 Å. The average value of the number of SBs was calculated as the ratio of the sum of the total number of SBs in each frame to the total number of frames. The SBs between the same two residues but different atoms were regarded as unique, keeping the one with the highest occupancy.

It is suggested that all C atoms within 3.9 Å interacts through hydrophobic contacts (Stojanovic and Zaric, 2009). We calculated the hydrophobic contacts between all the hydrophobic atoms (C, Cα, Cβ, Cδ, Cδ1, Cδ2, Cε, Cε1, Cε2, Cε3, Cγ, Cγ1, Cγ2, Cζ, Cζ2, Cζ3, and Cη2) with a cutoff of 4 Å, without redundancy. The structures were visualized by VMD and PyMOL (Schrödinger, 2010).

A one-way ANOVA was conducted here to evaluate whether the differences are significant for systems containing Ca^2+^, 318 K/298 K and 343 K/298 K, and systems without Ca^2+^ (318 K/298 K and 343 K/298 K) for the RMSD, *R*_g_, and SASA. The difference is considered significant in the case of *P* < 0.05.

## Results and Discussion

### Dynamics of Barley LD

**FIGURE 2:**
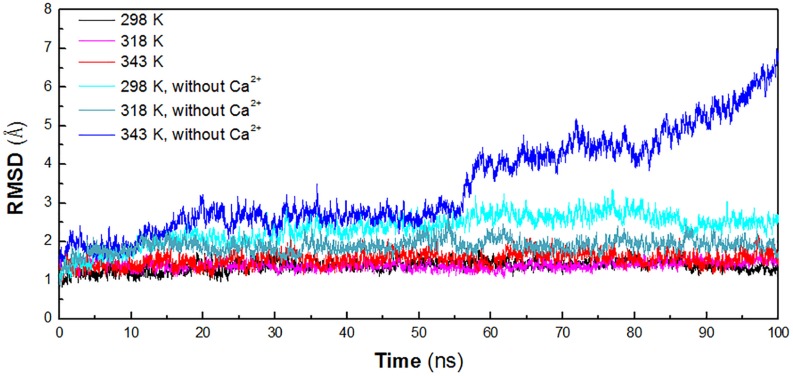
Time evolution of the backbone RMSD versus the starting structure of *Hv*LD. The simulation data obtained at 298, 318, 343, 298 K, without Ca^2+^, 318 K without Ca^2+^, and 343 K without Ca^2+^ are shown in black, magenta, red, cyan, deep teal, and blue, respectively.

In order to identify the key factors responsible for instability of *Hv*LD at high temperature, MD simulations were performed at different temperature conditions (298, 318, and 343 K) to predict the molecular behavior over the period of time using AMBER. To investigate the effect of calcium ions for the structural stability, MD simulations were also conducted for the three systems without Ca^2+^ (298, 318, and 343 K). The structural stability of six systems was examined by calculation of the RMSD of the backbone atoms relative to the initial structure. Figure 2 shows the RMSD variations of the six systems with respect to simulation time. It is observed that the systems are equilibrated and thus suitable for exploring the dynamics of *Hv*LD. The first three systems achieved equilibrium at 20, 10, and 25 ns, respectively. The RMSD values for the backbone atoms of *Hv*LD converge at 1.40 ± 0.11 and 1.39 ± 0.10 Å at 298 and 318 K and 1.59 ± 0.16 Å at 343 K. The statistical analysis suggests that the differences are significant (*P* < 0.05) (Supplementary Table S1). *Hv*LD in the systems without Ca^2+^ exhibits large variation compared with the initial structure at 298 and 343 K. The backbone RMSD increases rapidly and major structural distortion occurs at 343 K.

**FIGURE 3:**
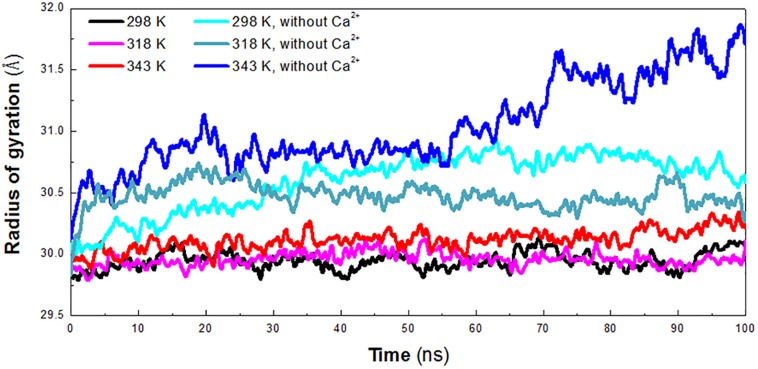
Radius of gyration (*R*_g_) plot. The simulation data obtained at 298 K, 318 K, 343 K, 298 K without Ca^2+^, 318 K without Ca^2+^, and 343 K without Ca^2+^ is shown in black, magenta, red, cyan, deep teal, and blue, respectively.

The radius of gyration (*R*_g_) reflects the compactness of protein structure. To detect the compactness of the overall structure, the radius of gyration (*R*_g_) of the protein in six systems was also calculated, as shown in Figure 3. According to RMSD plot, all the systems converged after 25 ns. We calculated the average *R*_g_ ranging from 25 to 100 ns in each system. The averaged *R*_g_ value indicated that the compactness of *Hv*LD increases when the temperature rises. *Hv*LD has the most compact structure at the lowest temperature (298 K, *R*_g_: 29.95 ± 0.09 Å). Moreover, *Hv*LD exhibits similar compactness at 318 K (*R*_g_: 29.97 ± 0.08 Å) and 343 K (*R*_g_: 30.14 ± 0.09 Å). The structure of *Hv*LD is less compact at the higher temperature than it at the lower temperature, indicating expansion of protein structure at higher temperature. The *Hv*LD exhibited higher *R*_g_ values at three systems without Ca^2+^, with the value of 30.69 ± 0.15 Å (298 K), 30.47 ± 0.10 (318 K), and 31.13 ± 0.34 Å (343 K), respectively. This result indicated that the structure of protein in the system without Ca^2+^ was less compact than those systems with Ca^2+^. In addition, with the temperature rising, the structure of *Hv*LD becomes less compact.

**FIGURE 4:**
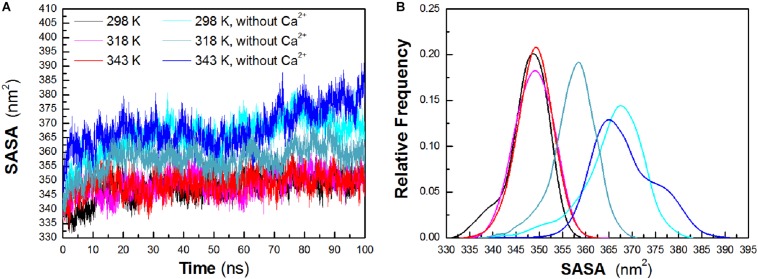
Total solvent-accessible surface area (SASA). **(A)** Time evolution of total SASA. **(B)** The distribution of total SASA. The simulation data obtained at 298, 318, 343, 298 K without Ca^2+^, 318 K without Ca^2+^, and 343 K without Ca^2+^ are shown in black, magenta, red, cyan, deep teal, and blue, respectively.

To evaluate the exposure of protein atoms to solvent, SASA was also obtained by calculating the surface area of atom in contact with solvent molecules. From Figure 4A, it is found that the total SASA values show a slight increase with a rise of temperature. The averaged SASA is 347.63 ± 4.29 nm^2^, 348.90 ± 3.95 nm^2^, and 349.05 ± 3.78 nm^2^ at 298 K, 318 K, and 343 K, respectively, while in the systems without Ca^2+^, the total SASA values exhibit significant increase at both temperatures. The averaged SASA is 365.80 ± 6.52 nm^2^, 357.43 ± 4.75 nm^2^, and 368.26 ± 6.79 nm^2^ at 298 K, 318 K, and 343 K, respectively. Total SASA, SASA of hydrophilic residues and catalytic residues, increases relatively from 298 K to 343 K (Table 1). The distribution of SASA is displayed in Figure 4B. It can be observed that the total SASA is from 331 to 359 nm^2^ at 298 K, with the major peak at 349 nm^2^ (20.73%). The same major peak appears at 318 K (18.94%) and 343 K (22.00%), respectively. In the systems without Ca^2+^, the total SASA increases dramatically. The range of SASA is from 337 to 383 nm^2^, with the major peak at 367 nm^2^ (14.91%) at 298 K. The range of SASA is from 337 to 371 nm^2^, with the major peak at 359 nm^2^ (18.95%) at 318 K. The SASA at 343 K distributes at a range of 339–391 nm^2^, with the major peak at 365 nm^2^ (13.31%). This profile is consistent with the trend of *R*_g_ values, which indicates that *Hv*LD become less compact with more solvent penetration into the core of the enzyme at high temperatures.

**TABLE 1:** Average value of SASA in different systems.

Systems	SASA_pho_	SASA_phil_	SASA_total_	SASA_catalytic_
298 K	110.48 ± 2.87	237.15 ± 2.52	347.63 ± 4.29	60.32 ± 7.83
318 K	111.56 ± 1.98	237.34 ± 2.81	348.90 ± 3.95	61.81 ± 6.90
343 K	110.05 ± 2.18	239.00 ± 2.81	349.05 ± 3.78	65.57 ± 7.34
298 K, no Ca^2+^	114.29 ± 3.16	251.51 ± 4.05	365.80 ± 6.52	67.34 ± 8.36
318 K, no Ca^2+^	111.15 ± 2.05	246.28 ± 3.30	357.43 ± 4.75	71.56 ± 9.25
343 K, no Ca^2+^	116.19 ± 2.67	252.08 ± 4.91	368.26 ± 6.79	71.88 ± 8.14

**FIGURE 5:**
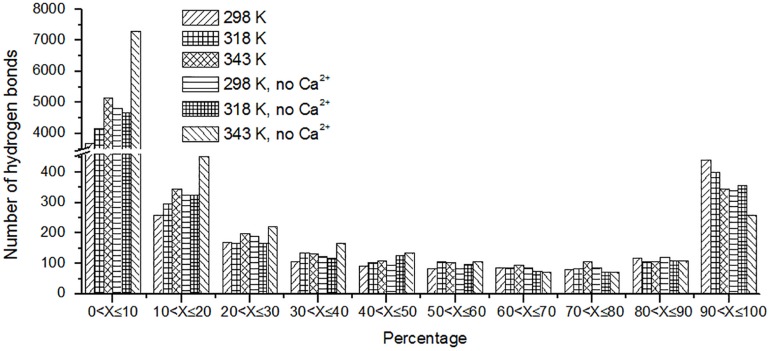
The number of hydrogen bonds (*y*-axis) based on percentage existence time (*x*-axis).

The hydrogen bond interaction is considered important in protein folding, stability, and function. It can be seen that *Hv*LD lost 55 hydrogen bonds at 343 K with respect to 298 K (Table 2). The number of hydrogen bonds also decreases in the systems without Ca^2+^. Based on the percentage existence time of HBs (Figure 5), short-lived HBs (0 < *X* ≤ 10%) increase significantly at 343 K, indicating that most of them appear transiently at high temperature. In contrast, the number of long-lived HBs decreases at 343 K, indicating that these interactions are unable to maintain at high temperature. In addition, the number of substantially live HBs (10 < *X* ≤ 90%) at 343 K is more than the corresponding value at 298 K, indicating that these HBs contribute to the stability of *Hv*LD. We analyzed the HBs with occupancy higher than 50% at 298, 318, and 343 K. There are 56 HBs becoming weak and their occupancy decreases with the increase in temperature (Supplementary Table S2). These HBs are very sensitive to temperature changes and they affect the thermostability of *Hv*LD.

**TABLE 2:** Average numbers of hydrogen bonds in different systems.

Systems	Total	MM	MS	SS	chr-chr	pho-pho	phi-phi	pho-phi
	HB	HB^a^	HB^b^	HB^c^	HB^d^	HB^e^	HB^f^	HB^g^
298 K	756.05	283.61	178.40	171.83	99.54	130.50	74.05	207.83
318 K	776.17	297.84	175.45	169.51	104.83	136.48	72.03	207.73
343 K	720.27	265.37	175.74	166.44	91.80	127.46	73.69	206.42
298 K, no Ca^2+^	754.33	295.91	178.82	150.73	81.55	140.52	74.21	204.34
318 K, no Ca^2+^	732.57	283.64	171.22	154.77	82.36	133.26	73.05	205.53
343 K, no Ca^2+^	745.39	287.05	174.38	155.38	90.66	138.89	66.77	195.73

*^*a*^Main chain–main chain hydrogen bonds. ^*b*^Main chain–side chain hydrogen bonds. ^*c*^Side chain–side chain hydrogen bonds. ^*d*^Hydrogen bonds between charged residues. ^*e*^Hydrogen bonds between hydrophilic residues. ^*f*^Hydrogen bonds between hydrophobic residues. ^*g*^Hydrogen bonds between hydrophilic residues and hydrophobic residues.*

Besides, we also analyzed the effect of temperature on main chain–main chain HBs, main chain–side chain HBs, and side chain–side chain HBs. There are more MM HBs compared with two other types. There are 18 MM HBs broken at 343 K compared with those at 298 K. MM HBs are important in secondary structure formation. The decrease of the number of MM HBs indicates that the stability of the secondary structure of the enzyme would be impaired. MS HBs and SS HBs exhibit slight decrease at 343 K with respect to 298 K. Moreover, HBs among the residues having similar/different chemical properties (charged–charged residues, hydrophobic–hydrophobic residues, hydrophilic–hydrophilic residues, and hydrophobic–hydrophilic residues) were analyzed. Among these, the number of HBs between hydrophilic–hydrophilic residues and hydrophobic–hydrophilic residues does not exhibit a significant difference at different temperatures, while the amount of HBs between charged–charged residues and hydrophobic–hydrophobic residues reduces at 343 K compared with that at 298 K. In the systems without Ca^2+^, the amount of the total HBs, MM HBs, MS HBs, and HBs for hydrophobic–hydrophobic residues, hydrophilic–hydrophilic residues, and hydrophobic–hydrophilic residues also decreases at 343 K compared with 298 K. The amount of SS HBs and HBs for charged–charged residues increases at 343 K compared with 298 K, indicating that there are new hydrogen bonds formed in the distorted structure.

The changes of HBs between protein and water were also explored in the temperature range of 298–343 K, which is displayed in Table 3. There is a decrease in protein–water HBs from 298 to 343 K, due to the loss of both M-water HBs and S-water HBs from 298 to 343 K. Besides, the number of HBs for hydrophobic residues to water, hydrophilic residues to water, and charged residues to water reduces at higher temperatures, indicating that the network of HBs between *Hv*LD and water molecules is broken. In the systems without Ca^2+^, the amount of total protein–water HBs and other types of protein–water HBs decreases at 343 K compared with 298 K, indicating the large change of the network of HBs between *Hv*LD and water molecules in these two systems.

**TABLE 3:** Average numbers of protein–water hydrogen bonds in different systems.

Systems	Pro-water	M-water	S-water	chr-water	pho-water	phi-water
	Total HB^a^	HB^b^	HB^c^	HB^d^	HB^e^	HB^f^
298 K	928.69	273.55	655.14	358.89	243.81	323.87
318 K	925.18	270.69	654.49	356.18	239.94	326.95
343 K	894.48	257.36	637.11	349.38	227.40	315.66
298 K, no Ca^2+^	967.29	275.80	691.49	390.55	245.21	329.83
318 K, no Ca^2+^	954.70	277.43	677.27	378.16	245.75	328.70
343 K, no Ca^2+^	924.28	262.96	661.31	363.69	237.24	321.18

*^*a*^Protein–water total hydrogen bond. ^*b*^Main chain–water hydrogen bond. ^*c*^Side chain–water hydrogen bond. ^*d*^Charge residues–water hydrogen bond. ^*e*^Hydrophilic residues–water hydrogen bond. ^*f*^Hydrophobic residues–water hydrogen bond.*

### The Stability of the Hydrogen Bond Between D403 and H404

**FIGURE 6:**
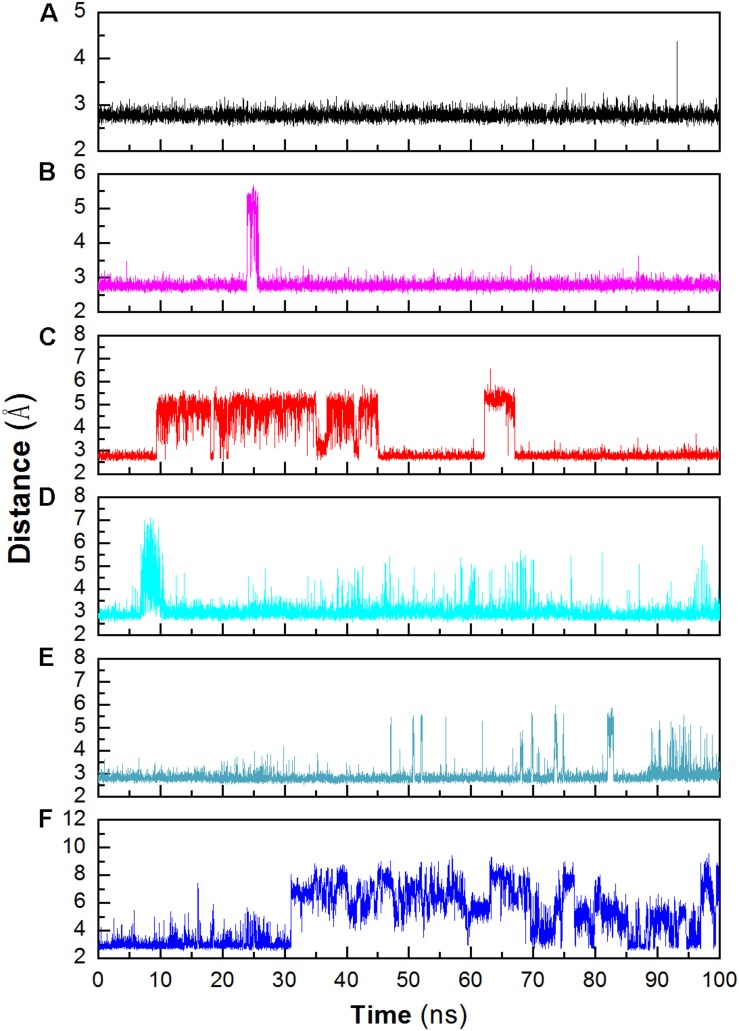
Monitoring the hydrogen bonds between the OD1 atom of Asp403 and the ND1 atom of His404 during MD simulation. **(A)** 298 K; **(B)** 318 K; **(C)** 343 K; **(D)** 298 K without Ca^2+^, **(E)** 318 K without Ca^2+^, **(F)** 343 K without Ca^2+^.

Structural stability may also affect the catalytic activity of this enzyme. The hydrogen bond between D403 and H404 is favorable for the stability of the catalytic triad, which is suggested in a previous work (Møller et al., 2015b). The distance between these two residues was monitored during the whole trajectory. It can be seen that the interaction is more stable at 298 and 318 K (Figure 6). When the temperature rises, the interaction becomes unstable (at 343 K). In addition, most of this interaction disappeared in the system without Ca^2+^ at 343 K. The unstable interaction would be unfavorable for the stability for the catalytic triad of Asp473–Glu510–Asp642.

### Salt Bridge Interaction Analysis

**FIGURE 7:**
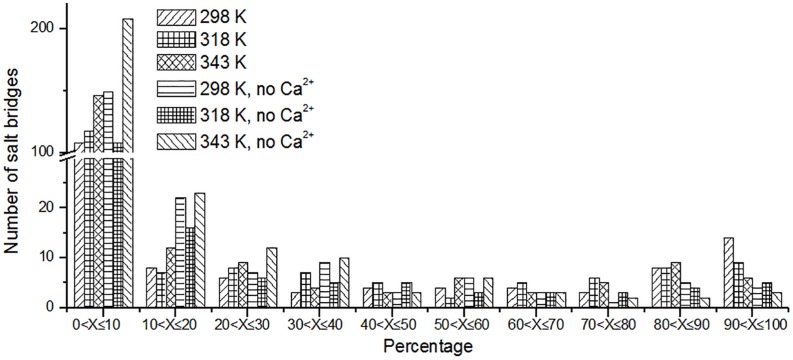
The number of unique salt bridges based on the percentage existence time at cutoff of 4 Å.

Besides hydrogen bonds, salt bridges are also very important for the stability of protein (Horovitz et al., 1990; Strop and Mayo, 2000; Jelesarov and Karshikoff, 2009). Salt bridges in enzyme may contribute to its stability at high temperature (Vieille and Zeikus, 2001; Kundu and Roy, 2010). Recently, Guo et al. (2018) summarized factors may contribute to the thermostability for pullulan-hydrolyzing enzymes. They found that there are more salt bridges in thermophilic pullulan-hydrolyzing enzymes than mesophilic ones, suggesting the importance of salt bridge for the enzyme pullulan-hydrolyzing thermostability. In this work, the salt bridges were identified using a 4-Å distance cutoff. We can observe that the number of short-lived salt bridges increases from 298 to 343 K (Figure 7), while the number of long-lived salt bridges decreases from 298 to 343 K. It is indicated that the short-lived salt bridges form transiently at high temperature. Some long-lived salt bridges are broken at high temperature. The number of long-lived salt bridges also decreases in the systems without Ca^2+^, which suggests that ions Ca^2+^ contribute to the stability of salt bridges.

We also analyzed the salt bridges with occupancy higher than 50%. The length of each salt bridge was averaged over the whole trajectory. There are 33, 30, and 29 salt bridges with occupancy higher than 50% during the simulations at 298, 318, and 343 K and 19 and 16 salt bridges in two systems without Ca^2+^ at 298 and 343 K. The salt bridges are noted to decrease in number with an increase in temperature. There are 21 common salt bridges identified at 298, 318, and 343 K. Among these salt bridges, five could still be maintained well even at high temperatures, suggesting an essential role in stabilizing this enzyme. There are eight salt bridges exhibiting comparable occupancy at three temperatures. These salt bridges were also not affected by increases in temperature. These 13 salt bridges are correlated with the partial structural stability of *Hv*LD, while 8 salt bridges become weak and their occupancy decreases with an increase in temperature (Table 4), including E568–R875, D317–R378, D803–R884, D457–R214, D468–R395, D456–R452, D399–R471, and D541–R542. These salt bridges are very sensitive to temperature changes and they affect the thermostability of *Hv*LD. All of the salt bridges are located on the surface of *Hv*LD.

**TABLE 4:** Average length and occupancy of important salt bridge interactions of *Hv*LD at 298, 318, and 343 K.

Salt bridge	298 K		318 K		343 K	
			
	Occupancy (%)	Distance (Å)	Occupancy (%)	Distance (Å)	Occupancy (%)	Distance (Å)
E568–R875	99.75	2.85 ± 0.17	81.06	3.28 ± 0.93	64.94	3.79 ± 1.12
D317–R378	99.19	2.84 ± 0.16	52.92	3.70 ± 0.62	59.23	3.55 ± 0.83
D803–R884	98.19	2.80 ± 0.12	92.80	2.82 ± 0.14	86.48	2.86 ± 0.20
D457–R214	97.66	2.88 ± 0.26	98.04	2.89 ± 0.26	73.57	3.35 ± 0.72
D468–R395	94.19	2.93 ± 0.25	91.04	2.96 ± 0.34	88.36	2.97 ± 0.32
D456–R452	94.10	3.06 ± 0.31	77.22	3.41 ± 0.92	57.88	3.75 ± 1.01
D399–R471	89.25	2.88 ± 0.24	88.84	2.85 ± 0.30	75.38	2.98 ± 0.34
D541–R542	84.31	3.11 ± 0.53	86.82	3.13 ± 0.44	67.38	3.63 ± 1.15

To evaluate the function of salt bridge, the salt bridge between Asp317 and Arg378 was selected. The MD simulations on LD^D317A^ at 298 and 343 K were conducted to evaluate the effect of salt bridge D317–R378 on the thermal stability of *Hv*LD. It is indicated that the break of this salt bridge destabilizes the protein. The LD^D317A^ exhibited higher RMSD, *R*_g_, and SASA than LD^WT^ at 298 K and 343 K (Supplementary Figure S1).

### Hydrophobic Contacts

**FIGURE 8:**
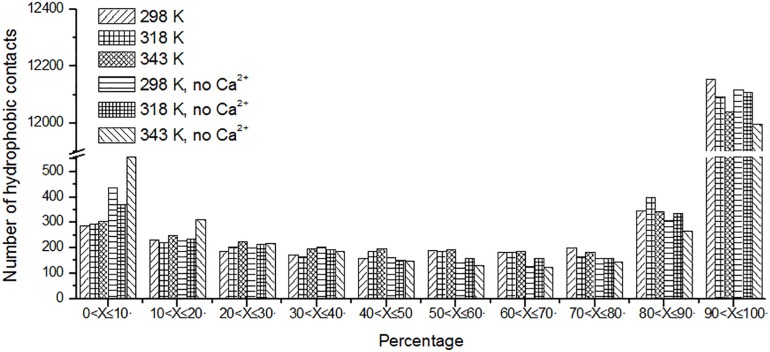
The number of unique hydrophobic contacts based on the percentage existence time at cutoff of 4 Å.

Protein stabilization is maintained by various interactions. Hydrophobic interaction is one of the important part of them (Van Dan Burg et al., 1994). Unique hydrophobic contacts were calculated as described in the “Materials and Methods” section. The percentage of time is displayed in Figure 8. It can be seen that the short-lived unique hydrophobic contacts (0 < *X* ≤ 10%) increase from 298 to 343 K. Long-lived hydrophobic contacts (90 < *X* ≤ 100%) decrease from 298 to 343 K. It exhibits the same trend with hydrogen bonds and salt bridges. A similar situation also occurs in systems without Ca^2+^.

### Identification of Thermal-Sensitive Regions

**FIGURE 9:**
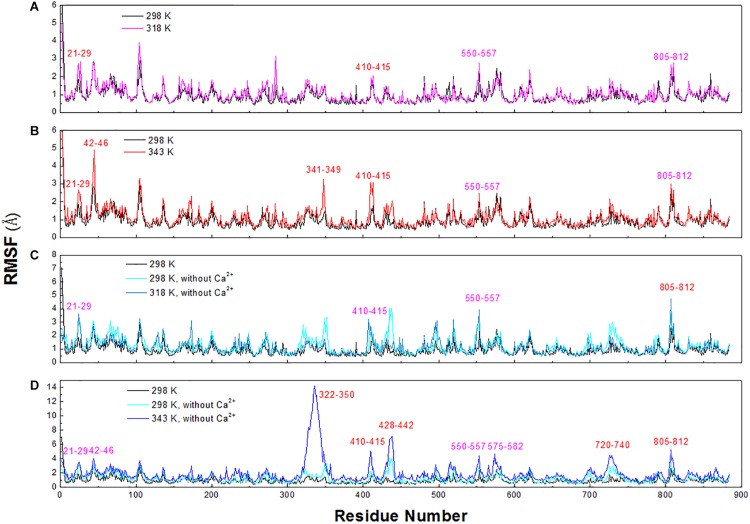
RMSF plots of the backbone atoms of six systems. **(A)** 298 and 318 K. **(B)** 298 and 343 K. **(C)** Simulation at 298 and 318 K without Ca^2+^. **(D)** Simulation at 298 and 343 K without Ca^2+^.

To explore the structural and dynamic changes, we identified the thermal-sensitive regions of *Hv*LD by analyzing the structural mobility based on the RMSF of the backbone atoms with respect to the initial structure. Figure 9 shows that the RMSF values of most regions of *Hv*LD fluctuate slightly at a temperature of up to 343 K, suggesting that these regions are relatively thermostable. Some regions showed steep RMSF fluctuations at high temperature, such as 318 and 343 K, indicating that those are thermal-sensitive regions. It can be observed in Figure 9 that the highest fluctuations occur at the N-terminal because it was not restrained. Also, some loops that always exhibit high fluctuation at different temperatures may be due to their intrinsic flexibility, including residues 102–107, 135–138, and 575–582. Besides, regions that fluctuated higher than 0.5 and 1.0 Å are highlighted by magenta and red in Figure 9. Larger fluctuations are observed at 318 K or 343 K, such as residues 21–29, 42–46, 341–349, and 410–415. These regions are predicted to be thermal-sensitive regions. In a previous work, it was suggested that residues 23–27, 42–48, 102–109, and 806–810 exhibited high flexibility, with low level electron density (Vester-Christensen et al., 2010), which is consistent with our results. To compare with those systems without Ca^2+^, it can be observed that the flexibility of LD is all higher in these systems than that at 298 K. In particular, these loops exhibit high fluctuations, including residues 21–29, 42–46, 322–350, 410–415, 428–442, 550–557, 575–582, 720–740, and 805–812.

**FIGURE 10:**
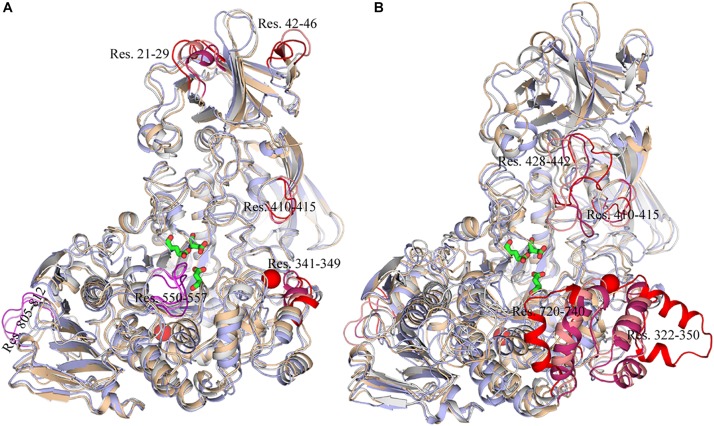
Snapshots of *Hv*LD structures from MD trajectories. **(A)** Comparison of the structure extracted from the system at 298, 318, and 343 K, which are colored by white, wheat, and light blue, respectively. **(B)** Comparison of the structure extracted from the system at 298, 298, and 343 K without Ca^2+^, which are colored by white, wheat, and light blue, respectively. The high flexible region was colored by red.

To exhibit these regions in *Hv*LD, the higher fluctuation regions were mapped onto the tertiary structure. As shown in Figure 10, it is observed that some of them are located near the catalytic crevice, including residues 550–557 (Figure 10A). The high flexibility of these regions would decrease the stability of the catalytic triad. Residues 21–29, 42–46, 341–349, and 410–415 are located in surface loops (Figure 10A). In the systems without Ca^2+^, there are large conformational change of residues 322–350, 428–442, 720–740, and 805–812 at high temperature (343 K) (Figure 10B). The loss of Ca^2+^ made the significant conformational change of a long loop in catalytic (β/α) eight domain, previously described as loop 2 (Jespersen et al., 1991; Mikami et al., 2006; Turkenburg et al., 2009) (the site for Ca1) (Supplementary Figure S2), indicating that this Ca^2+^ are important for maintaining the stable conformation of *Hv*LD at high temperature. The conformational change of loop 2 affected the conformation of residue 322–350 and 720–740. The removal of another Ca^2+^, which is located between α1 and α2, had a weaker effect on the conformation of *Hv*LD at different temperatures. This Ca^2+^ contributes less to the stability of *Hv*LD.

## Conclusion

In the present study, thermostability factors of barley LD were investigated by MD simulations. The higher value of RMSD, *R*_g_, and SASA suggests the instability of *Hv*LD at high temperatures. Intra-protein hydrogen bonds and hydrogen bonds between protein and water decrease at high temperature. Long-lived hydrogen bonds, salt bridges, and hydrophobic contacts are lost at high temperature. The salt bridge interaction analysis suggests that these salt bridges are important for the thermostability of *Hv*LD, including E568–R875, D317–R378, D803–R884, D457–R214, D468–R395, D456–R452, D399–R471, and D541–R542, which are located on the surface of *Hv*LD. Based on RMSF calculations for *Hv*LD at various temperatures, several thermally sensitive regions of *Hv*LD were identified, such as residues 21–29, 42–46, 341–349, and 410–415. The structural and dynamic details will help to understand the driving forces that lead to the stability of *Hv*LD at different temperatures, which will facilitate enzyme engineering of *Hv*LD for enhanced thermostability.

## Data Availability Statement

The raw data supporting the conclusions of this article will be made available by the authors, without undue reservation, to any qualified researcher.

## Author Contributions

JDu conceived and supervised the experiments. JDo, SD, and KZ performed the MD simulations. JDo, JDu, JY, and SH analyzed the data. JDu and HY wrote the manuscript.

## Conflict of Interest

JDu, JDo, JY, SH, and HY were employed by company Tsingtao Brewery. The remaining authors declare that the research was conducted in the absence of any commercial or financial relationships that could be construed as a potential conflict of interest.

## Supplementary Material

FIGURE S1 RMSD, *R*_g_, and SASA for HvLD^D317A^. **(A)** RMSD plot. **(B)** *R*_g_ plot. **(C)** SASA plot.

FIGURE S2 The conformation of Ca1 site. Initial structure and the extracted structure form system without Ca^2+^ at 343 K are colored by cyan and purple, respectively.

TABLE S1 Statistical analysis results of RMSD, *R*_g_ and SASA for HvLD at different simulation temperatures.

TABLE S2 Length and occupancy of important hydrogen bond interactions of HvLD at 298, 318, and 343 K.

## References

[B1] Alizadeh-Rahrovi J. Shayesteh A. Ebrahim-Habibi A. (2015). Structural stability of myoglobin and glycomyoglobin: a comparative molecular dynamics simulation study. *J. Biol. Phys.* 41 349–366. 10.1007/s10867-015-9383-2 25701404 PMC4550620

[B2] Anandakrishnan R. Aguilar B. Onufriev A. V. (2012). H++ 3.0: automating pK prediction and the preparation of biomolecular structures for atomistic molecular modeling and simulations. *Nucleic Acids Res.* 40 W537–W541. 10.1093/nar/gks375 22570416 PMC3394296

[B3] Bradbrook G. M. Gleichmann T. Harrop S. J. Habash J. Raftery J. Kalb J. (1998). X-Ray and molecular dynamics studies of concanavalin-A glucoside and mannoside complexes Relating structure to thermodynamics of binding. *J. Chem. Soc. Faraday Trans.* 94 1603–1611. 10.1039/A800429C

[B4] Chang M. Chu X. Lv J. Li Q. Tian J. Wu N. (2016). Improving the thermostability of acidic pullulanase from bacillus naganoensis by rational design. *PLoS One* 11:e0165006. 10.1371/journal.pone.0165006 27764201 PMC5072709

[B5] Chen A. Li Y. Nie J. McNeil B. Jeffrey L. Yang Y. (2015). Protein engineering of Bacillus acidopullulyticus pullulanase for enhanced thermostability using in silico data driven rational design methods. *Enzyme Microb. Technol.* 78 74–83. 10.1016/j.enzmictec.2015.06.013 26215347

[B6] Coleman T. G. Mesick H. C. Darby R. L. (1977). Numerical integration: a method for improving solution stability in models of the circulation. *Ann. Biomed. Eng.* 5 322–328. 10.1007/BF02367312 607820

[B7] Darden T. York D. Pedersen L. (1993). Particle mesh Ewald: An N⋅log(N) method for Ewald sums in large systems. *J. Chem.Phys.* 98 10089–10092. 10.1063/1.464397

[B8] Gu J. Tong H. Sun L. Lin Z. (2019). Molecular dynamics perspective on the thermal stability of mandelate racemase. *J. Biomol. Struct. Dyn.* 37 383–393. 10.1080/07391102.2018.1427631 29334318

[B9] Guo J. Coker A. R. Wood S. P. Cooper J. B. Keegan R. M. Ahmad N. (2018). Structure and function of the type III pullulan hydrolase from Thermococcus kodakarensis. *Acta Crystallogr. Sec. D* 74 305–314. 10.1107/S2059798318001754 29652257

[B10] Hornak V. Abel R. Okur A. Strockbine B. Roitberg A. Simmerling C. (2006). Comparison of multiple Amber force fields and development of improved protein backbone parameters. *Proteins* 65 712–725. 10.1002/prot.21123 16981200 PMC4805110

[B11] Horovitz A. Serrano L. Avron B. Bycroft M. Fersht A. R. (1990). Strength and co-operativity of contributions of surface salt bridges to protein stability. *J. Mol. Biol.* 216 1031–1044. 10.1016/S0022-2836(99)80018-7 2266554

[B12] Humphrey W. Dalke A. Schulten K. (1996). VMD: Visual molecular dynamics. *J. Mol. Graph.* 14 33–38. 10.1016/0263-7855(96)00018-5 8744570

[B13] Idrees D. Rahman S. Shahbaaz M. Haque M. A. Islam A. Ahmad F. (2017). Estimation of thermodynamic stability of human carbonic anhydrase IX from urea-induced denaturation and MD simulation studies. *Int. J. Biol. Macromol.* 105 183–189. 10.1016/j.ijbiomac.2017.07.010 28688947

[B14] Jelesarov I. Karshikoff A. (2009). “Defining the role of salt bridges in protein stability,” in *Protein Structure, Stability, and Interactions*, ed. Shriver J. W. (Totowa, NJ: Humana Press), 10.1007/978-1-59745-367-7_10 19157086

[B15] Jespersen H. M. MacGregor E. A. Sierks M. R. Svensson B. (1991). Comparison of the domain-level organization of starch hydrolases and related enzymes. *Biochem. J.* 280 51–55. 10.1042/bj2800051 1741756 PMC1130598

[B16] Jiang X. Chen G. Wang L. (2016). Structural and dynamic evolution of the amphipathic N-terminus diversifies enzyme thermostability in the glycoside hydrolase family 12. *Phys. Chem. Chem. Phys.* 18 21340–21350. 10.1039/C6CP02998A 27425569

[B17] Jorgensen W. L. Chandrasekhar J. Madura J. D. Impey R. W. Klein M. L. (1983). Comparison of simple potential functions for simulating liquid water. *J. Chem. Phys.* 79 926–935. 10.1063/1.445869

[B18] Kundu S. Roy D. (2010). Structural study of carboxylesterase from hyperthermophilic bacteria Geobacillus stearothermophilus by molecular dynamics simulation. *J. Mol. Graph. Model.* 28 820–827. 10.1016/j.jmgm.2010.03.001 20347362

[B19] Li S. Xu J. Bao Y. Zheng H. Song H. (2015). Structure and sequence analysis-based engineering of pullulanase from Anoxybacillus sp. LM18-11 for improved thermostability. *J. Biotechnol.* 210 8–14. 10.1016/j.jbiotec.2015.06.406 26116135

[B20] Manners D. J. Marshall J. J. Yellowlees D. (1970). The specificity of cereal limit dextrinases. *Biochem. J.* 116 539–541. 10.1042/bj1160539 5435695 PMC1185392

[B21] Mikami B. Iwamoto H. Malle D. Yoon H.-J. Demirkan-Sarikaya E. Mezaki Y. (2006). Crystal structure of pullulanase: evidence for parallel binding of oligosaccharides in the active site. *J. Mol. Biol.* 359 690–707. 10.1016/j.jmb.2006.03.058 16650854

[B22] Møller M. Hachem M. Abou Svensson B. Henriksen A. (2012a). Structure of the starch-debranching enzyme barley limit dextrinase reveals homology of the N-terminal domain to CBM21. *Acta Crystallogr. Sect. F Struct. Biol. Cryst. Commun.* 68(Pt 9), 1008–1012. 10.1107/S1744309112031004 22949184 PMC3433187

[B23] Møller M. S. Abou Hachem M. Svensson B. Henriksen A. (2012b). Structure of the starch-debranching enzyme barley limit dextrinase reveals homology of the N-terminal domain to CBM21. *Acta Crystallogr.* 68(Pt 9), 1008–1012. 10.1107/S1744309112031004 PMC3433187 22949184

[B24] Møller M. S. Vester-Christensen M. B. Jensen J. M. Hachem M. A. Henriksen A. Svensson B. (2015a). Crystal Structure of Barley Limit Dextrinase-Limit Dextrinase Inhibitor (LD-LDI) Complex Reveals Insights into Mechanism and Diversity of Cereal Type Inhibitors. *J. Biol. Chem.* 290 12614–12629. 10.1074/jbc.M115.642777 25792743 PMC4432282

[B25] Møller M. S. Windahl M. S. Sim L. Bøjstrup M. Abou Hachem M. Hindsgaul O. (2015b). Oligosaccharide and Substrate Binding in the Starch Debranching Enzyme Barley Limit Dextrinase. *J. Mol. Biol.* 427(6 Pt B), 1263–1277. 10.1016/j.jmb.2014.12.019 25562209

[B26] Moshi A. P. Hosea K. M. M. Elisante E. Mamo G. Mattiasson B. (2015). High temperature simultaneous saccharification and fermentation of starch from inedible wild cassava (Manihot glaziovii) to bioethanol using *Caloramator boliviensis*. *Bioresour. Technol.* 180 128–136. 10.1016/j.biortech.2014.12.087 25594508

[B27] Nick Pace C. Scholtz J. M. Grimsley G. R. (2014). Forces stabilizing proteins. *FEBS Lett.* 588 2177–2184. 10.1016/j.febslet.2014.05.006 24846139 PMC4116631

[B28] Nilofer C. Sukhwal A. Mohanapriya A. Kangueane P. (2017). Protein-protein interfaces are vdW dominant with selective H-bonds and (or) electrostatics towards broad functional specificity. *Bioinformation* 13 164–173. 10.6026/97320630013164 28729757 PMC5512853

[B29] Roe D. R. Cheatham T. E. (2013). PTRAJ and CPPTRAJ: software for processing and analysis of molecular dynamics trajectory data. *J. Chem. Theor. Comput.* 9 3084–3095. 10.1021/ct400341p 26583988

[B30] Schrödinger L. L. C. (2010). *The PyMOL Molecular Graphics System, Version 1.3.1. The PyMOL Molecular Graphics System, Version 1.3.1.*

[B31] Sharma R. Sastry G. N. (2015). Deciphering the dynamics of non-covalent interactions affecting thermal stability of a protein: molecular dynamics study on point mutant of thermus thermophilus isopropylmalate dehydrogenase. *PLoS One* 10:e0144294. 10.1371/journal.pone.0144294 26657745 PMC4689552

[B32] Sissons M. Taylor M. Proudlove M. (1995). Barley malt limit dextrinase: Its extraction, heat stability, and activity during malting and mashing. *Am. Soc. Brew. Chem.* 21 S356.

[B33] Sissons M. J. Lance R. C. M. Wallace W. (1994). Bound and free forms of barley limit dextrinase. *Cereal Chem.* 71 520–521.

[B34] Stam M. R. Danchin E. G. J. Rancurel C. Coutinho P. M. Henrissat B. (2006). Dividing the large glycoside hydrolase family 13 into subfamilies: towards improved functional annotations of α-amylase-related proteins. *Protein Eng. Design Select.* 19 555–562. 10.1093/protein/gzl044 17085431

[B35] Stojanovic S. D. Zaric S. D. (2009). Hydrogen bonds and hydrophobic interactions of porphyrins in porphyrin-containing proteins. *Open Struct. Biol. J.* 3 34–41. 10.2174/1874199100903010034

[B36] Strop P. Mayo S. L. (2000). Contribution of surface salt bridges to protein stability. *Biochemistry* 39 1251–1255. 10.1021/bi992257j 10684603

[B37] Turkenburg J. P. Brzozowski A. M. Svendsen A. Borchert T. V. Davies G. J. Wilson K. S. (2009). Structure of a pullulanase from Bacillus acidopullulyticus. *Proteins* 76 516–519. 10.1002/prot.22416 19382205

[B38] Van Dan Burg B. Dijkstra B. W. Vriend G. Van Dar Vinne B. Venema G. Eijsink V. G. H. (1994). Protein stabilization by hydrophobic interactions at the surface. *Eur. J. Biochem.* 220 981–985. 10.1111/j.1432-1033.1994.tb18702.x 8143751

[B39] Vester-Christensen M. B. Hachem M. A. Svensson B. Henriksen A. (2010). Crystal structure of an essential enzyme in seed starch degradation: barley limit dextrinase in complex with cyclodextrins. *J. Mol. Biol.* 403 739–750. 10.1016/j.jmb.2010.09.031 20863834

[B40] Vieille C. Zeikus G. J. (2001). Hyperthermophilic enzymes: sources, uses, and molecular mechanisms for thermostability. *Microbiol. Mol. Biol. Rev.* 65 1–43. 10.1128/mmbr.65.1.1-43.2001 11238984 PMC99017

[B41] Wang J. Wolf R. M. Caldwell J. W. Kollman P. A. Case D. A. (2004). Development and testing of a general amber force field. *J. Comput. Chem.* 25 1157–1174. 10.1002/jcc.20035 15116359

[B42] Wang X. Nie Y. Mu X. Xu Y. Xiao R. (2016). Disorder prediction-based construct optimization improves activity and catalytic efficiency of Bacillus naganoensis pullulanase. *Sci. Rep.* 6:24574. 10.1038/srep24574 27091115 PMC4835747

[B43] Wang X. Zhang X. Cai S. Ye L. Zhou M. Chen Z. (2015). Genetic diversity and QTL mapping of thermostability of limit dextrinase in barley. *J. Agric. Food Chem.* 63 3778–3783. 10.1021/acs.jafc.5b00190 25816850

[B44] Yang X. Westcott S. Gong X. Evans E. Zhang X.-Q. Lance R. C. M. (2008). Amino acid substitutions of the limit dextrinase gene in barley are associated with enzyme thermostability. *Mol. Breed.* 23 61 10.1007/s11032-008-9214-2

