# Corrigendum: Understanding Thermostability Factors of Barley Limit Dextrinase by Molecular Dynamics Simulations

**DOI:** 10.3389/fmolb.2020.00123

**Published:** 2020-07-07

**Authors:** Juan Du, Jianjun Dong, Songjie Du, Kun Zhang, Junhong Yu, Shumin Hu, Hua Yin

**Affiliations:** ^1^State Key Laboratory of Biological Fermentation Engineering of Beer, Tsingtao Brewery, Qingdao, China; ^2^Shandong Province Key Laboratory of Applied Mycology, College of Life Sciences, Qingdao Agricultural University, Qingdao, China

**Keywords:** barley limit dextrinase, thermostability, molecular dynamics simulation, hydrogen bond, salt-bridge

In the original article, there was an error in affiliation 1. Instead of “State Key Laboratory of Biological Fermentation Engineering of Beer, College of Life Sciences, Qingdao Agricultural University, Qingdao, China,” it should be “State Key Laboratory of Biological Fermentation Engineering of Beer, Tsingtao Brewery, Qingdao, China.”

The authors apologize for this error and state that this does not change the scientific conclusions of the article in any way. The original article has been updated.

## Conflict of Interest

JDu, JDo, JY, SH, and HY were employed by company Tsingtao Brewery. The remaining authors declare that the research was conducted in the absence of any commercial or financial relationships that could be construed as a potential conflict of interest.

