# Supplementary figures and images for: Understanding Thermostability Factors of Barley Limit Dextrinase by Molecular Dynamics Simulations
Source: Front Mol Biosci. 2020 Apr 16;7:51. doi: 10.3389/fmolb.2020.00051 (PMC7241666; doi:10.3389/fmolb.2020.00051)

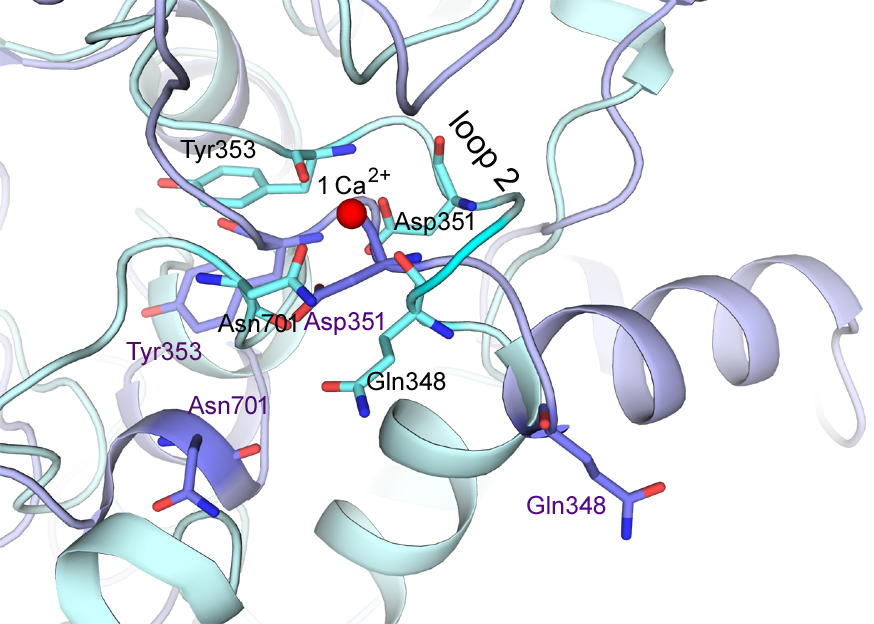

Supplement: FIGURE S2 — The conformation of Ca1 site. Initial structure and the extracted structure form system without Ca2+ at 343 K are colored by cyan and purple, respectively. [file Image_2.tif]
