# Supplementary material for: Understanding Thermostability Factors of Barley Limit Dextrinase by Molecular Dynamics Simulations
Source: Front Mol Biosci. 2020 Apr 16;7:51. doi: 10.3389/fmolb.2020.00051 (PMC7241666; doi:10.3389/fmolb.2020.00051)
Supplement: TABLE S1 — Statistical analysis results of RMSD, Rg and SASA for HvLD at different simulation temperatures. [file Table_1.DOCX]

Table S1 Statistical analysis results of RMSD, Rg and SASA for HvLD at different temperatures

|  | 318 K / 298 K | | | 343 K / 298 K | | |
| --- | --- | --- | --- | --- | --- | --- |
|  | SS | F | P-value | SS | F | P-value |
| RMSD | 2.53 | 169.29 | 1.5093E-38 | 159.38 | 6487.75 | 0 |
| Rg | 1.01 | 134.13 | 6.4376E-31 | 147.65 | 15301.80 | 0 |
| SASA | 8.09E+07 | 474.79 | 4.70E-104 | 1.01E+08 | 616.26 | 5E-134 |
|  | 318 K / 298 K (system without Ca^2+^) | | | 343 K / 298 K (system without Ca^2+^) | | |
|  | SS | F | P-value | SS | F | P-value |
| RMSD | 1021.63 | 11205.34 | 0 | 6294.85 | 7388.65 | 0 |
| Rg | 73.84 | 2066.27 | 0 | 988.23 | 10377.55 | 0 |
| SASA | 3.50E+09 | 10770.46 | 0 | 3.03E+08 | 682.94 | 5E-148 |

Table S2 Length and occupancy of important hydrogen bond interactions of HvLD at 298K, 318K, and 343K.

| Hydrogen Bond | | 298 K | | | 318 K | | | 343 K | | |
| --- | --- | --- | --- | --- | --- | --- | --- | --- | --- | --- |
|  |  | Occupancy (%) | Distance (Å) | Angle (˚) | Occupancy (%) | Distance (Å) | Angle (˚) | Occupancy (%) | Distance (Å) | Angle (˚) |
| [ASP815@OD1](mailto:ASP815@OD1) | ASN817@N | 99.64 | 2.90 | 160.53 | 59.00 | 2.91 | 159.39 | 55.80 | 2.93 | 158.48 |
| ASN403@OD1 | HIS404@ND1 | 99.92 | 2.77 | 155.77 | 98.14 | 2.78 | 156.26 | 60.10 | 2.78 | 158.18 |
| GLU435@O | ASN442@N | 99.80 | 2.94 | 161.04 | 99.58 | 2.99 | 160.21 | 66.91 | 3.02 | 160.56 |
| ASN442@OD1 | ALA438@N | 99.73 | 3.00 | 160.67 | 99.73 | 2.98 | 159.82 | 58.25 | 2.99 | 157.95 |
| GLU381@OE2 | SER371@OG | 99.73 | 2.63 | 163.51 | 54.29 | 2.76 | 159.59 | 53.52 | 2.74 | 160.23 |
| ARG542@O | GLN590@NE2 | 99.69 | 2.89 | 161.43 | 98.48 | 2.90 | 162.03 | 85.52 | 2.90 | 160.48 |
| GLU516@OE2 | ASN442@ND2 | 99.57 | 2.85 | 160.49 | 99.93 | 2.83 | 161.84 | 58.33 | 2.84 | 159.19 |
| SER677@OG | ASN636@ND2 | 99.28 | 2.94 | 159.36 | 98.34 | 2.95 | 154.67 | 86.32 | 3.00 | 155.46 |
| THR580@O | LEU584@N | 99.20 | 2.97 | 161.67 | 91.60 | 3.04 | 159.93 | 87.06 | 3.07 | 160.50 |
| SER135@O | ARG182@NH1 | 99.17 | 2.84 | 155.81 | 98.44 | 2.85 | 155.61 | 85.04 | 2.86 | 154.52 |
| VAL175@O | SER172@N | 99.12 | 2.94 | 157.52 | 98.40 | 2.96 | 156.99 | 83.38 | 2.96 | 156.13 |
| GLU381@OE1 | SER371@N | 99.08 | 2.83 | 152.28 | 81.14 | 2.97 | 149.06 | 50.14 | 2.96 | 148.46 |
| ASP338@O | GLN341@NE2 | 98.99 | 2.92 | 158.44 | 97.47 | 2.92 | 158.44 | 75.88 | 2.94 | 158.67 |
| ASN436@OD1 | ASN441@N | 98.75 | 2.96 | 161.38 | 97.25 | 2.98 | 160.82 | 57.25 | 2.96 | 157.94 |
| VAL798@O | HIS786@N | 98.74 | 2.96 | 160.68 | 97.99 | 2.98 | 160.64 | 73.82 | 3.09 | 156.43 |
| ILE261@O | SER265@OG | 98.59 | 2.69 | 163.25 | 92.60 | 2.79 | 157.84 | 86.00 | 2.81 | 155.99 |
| SER172@O | VAL175@N | 98.45 | 3.01 | 153.17 | 96.97 | 3.02 | 152.93 | 81.29 | 3.04 | 153.04 |
| ILE764@O | SER767@OG | 98.37 | 2.68 | 165.21 | 95.99 | 2.70 | 164.89 | 83.67 | 3.09 | 146.40 |
| GLU568@O | GLN559@NE2 | 98.28 | 2.97 | 156.83 | 95.22 | 2.95 | 158.85 | 84.78 | 2.95 | 156.77 |
| GLY375@O | SER215@OG | 98.05 | 2.75 | 155.90 | 82.78 | 2.79 | 153.48 | 77.10 | 2.80 | 151.70 |
| SER437@OG | ASN442@ND2 | 97.88 | 2.91 | 144.07 | 97.67 | 2.93 | 142.22 | 56.76 | 2.91 | 144.45 |
| PRO334@O | SER337@N | 97.47 | 3.07 | 155.51 | 89.86 | 3.12 | 154.30 | 81.04 | 3.15 | 152.90 |
| SER177@O | LYS170@N | 97.34 | 3.03 | 161.69 | 95.85 | 3.04 | 161.33 | 84.07 | 3.03 | 160.41 |
| ILE347@O | GLU350@N | 97.21 | 2.97 | 150.56 | 96.74 | 3.01 | 153.41 | 57.70 | 3.05 | 151.63 |
| SER767@OG | HIS847@NE2 | 96.79 | 2.91 | 148.12 | 92.92 | 2.94 | 147.11 | 86.98 | 2.98 | 147.53 |
| PRO624@O | ARG542@NE | 95.94 | 3.00 | 145.57 | 93.58 | 3.05 | 142.67 | 72.97 | 3.01 | 145.36 |
| GLN341@OE1 | SER725@N | 95.88 | 3.04 | 161.43 | 93.57 | 3.03 | 162.79 | 77.81 | 3.04 | 160.08 |
| ASP648@O | VAL652@N | 95.78 | 2.94 | 146.47 | 81.48 | 3.03 | 142.31 | 77.18 | 3.07 | 145.86 |
| GLY409@O | SER415@OG | 95.70 | 2.77 | 160.97 | 89.03 | 2.79 | 162.50 | 62.95 | 2.78 | 161.71 |
| HIS292@O | LEU296@N | 95.51 | 3.08 | 156.09 | 87.77 | 3.12 | 155.52 | 76.51 | 3.18 | 154.86 |
| THR586@O | GLN590@N | 95.42 | 3.09 | 158.15 | 82.83 | 3.17 | 157.25 | 78.41 | 3.17 | 158.96 |
| GLY356@O | ASN358@N | 94.62 | 2.90 | 146.37 | 90.84 | 2.91 | 146.48 | 72.20 | 2.97 | 139.30 |
| ASP760@OD2 | SER853@OG | 94.27 | 2.67 | 164.69 | 93.47 | 2.67 | 164.73 | 60.19 | 2.65 | 164.47 |
| ASN525@O | ASN530@ND2 | 94.21 | 2.93 | 158.26 | 85.46 | 2.94 | 156.72 | 70.85 | 2.95 | 158.36 |
| MET674@O | SER677@N | 94.17 | 3.15 | 154.48 | 82.30 | 3.20 | 153.22 | 79.44 | 2.79 | 158.89 |
| ASN576@O | THR580@OG1 | 91.45 | 2.78 | 161.05 | 85.58 | 2.81 | 160.77 | 65.67 | 2.88 | 160.03 |
| VAL344@O | ILE347@N | 91.39 | 3.06 | 152.98 | 81.95 | 3.04 | 152.21 | 66.14 | 3.12 | 152.97 |
| PRO631@O | THR634@N | 89.15 | 3.16 | 154.64 | 84.77 | 3.17 | 155.36 | 71.77 | 3.19 | 155.88 |
| GLY22@O | ALA26@N | 89.06 | 3.02 | 145.49 | 79.64 | 2.98 | 143.55 | 70.58 | 3.03 | 147.04 |
| TRP461@O | VAL467@N | 87.95 | 3.17 | 163.54 | 74.10 | 3.21 | 163.28 | 64.58 | 3.22 | 162.51 |
| ASP648@OD2 | SER692@OG | 87.91 | 2.60 | 164.97 | 74.05 | 2.80 | 152.76 | 68.23 | 2.81 | 152.39 |
| LEU120@O | PHE124@N | 87.56 | 3.07 | 140.16 | 78.69 | 3.11 | 139.41 | 67.77 | 3.13 | 138.46 |
| GLN340@O | VAL344@N | 85.08 | 3.16 | 154.75 | 63.27 | 3.19 | 154.74 | 55.77 | 3.19 | 153.50 |
| MET339@O | ALA343@N | 84.69 | 3.12 | 151.41 | 73.38 | 3.12 | 150.89 | 68.99 | 3.12 | 150.17 |
| ASP110@OD1 | VAL111@N | 84.21 | 2.88 | 135.37 | 78.35 | 2.87 | 135.78 | 65.01 | 2.87 | 135.84 |
| LYS295@O | ALA299@N | 84.06 | 3.10 | 144.60 | 71.64 | 3.15 | 143.60 | 66.44 | 3.16 | 144.11 |
| GLN590@O | GLY594@N | 82.82 | 3.14 | 155.00 | 79.68 | 3.18 | 155.67 | 56.01 | 3.21 | 148.13 |
| LEU216@O | GLY375@N | 78.95 | 3.10 | 152.24 | 61.00 | 3.13 | 152.66 | 55.24 | 3.12 | 149.77 |
| LEU405@O | ASN441@ND2 | 78.21 | 3.08 | 161.17 | 77.33 | 3.05 | 160.91 | 56.32 | 3.07 | 156.86 |
| LEU241@O | GLU244@N | 78.00 | 2.99 | 140.63 | 67.10 | 3.02 | 138.79 | 64.03 | 3.01 | 140.04 |
| TRP705@O | ASN717@ND2 | 76.45 | 2.98 | 159.69 | 67.78 | 2.96 | 159.39 | 56.92 | 3.02 | 159.62 |
| GLU231@O | LYS234@N | 70.36 | 3.21 | 153.95 | 68.61 | 3.19 | 154.28 | 56.52 | 3.21 | 153.70 |
| ASP457@O | ASN460@ND2 | 70.31 | 3.15 | 153.96 | 59.44 | 3.14 | 156.34 | 58.91 | 3.12 | 155.28 |
| ASN441@OD1 | SER408@OG | 69.89 | 2.78 | 158.96 | 58.30 | 2.82 | 156.76 | 51.07 | 2.78 | 159.05 |
| ASP815@OD2 | SER768@OG | 69.65 | 2.91 | 136.65 | 58.57 | 2.64 | 165.63 | 54.11 | 2.65 | 165.13 |
| ASP710@OD2 | THR712@OG1 | 66.96 | 2.87 | 154.91 | 53.49 | 2.99 | 151.86 | 50.12 | 3.01 | 153.45 |
